# Social interaction is associated with changes in infants’ motor activity

**DOI:** 10.3402/snp.v5.28256

**Published:** 2015-11-05

**Authors:** Céline Scola, Marie Bourjade, Marianne Jover

**Affiliations:** 1 Aix Marseille Université, PSYCLE EA 3273, 13621, Aix-en-Provence, France; 2 ‘Cognition, Communication & Development’ Team, Octogone Interdisciplinary Research Unit, University of Toulouse Jean Jaurès, Toulouse, France; 3 Aix Marseille Université, CNRS, LPL UMR 7309, 13100, Aix-en-Provence, France

**Keywords:** mother–child interaction, motor activity, infant, context

## Abstract

**Background:**

In developmental research, infants are commonly assumed to be early stakeholders in interactions with their caregivers. The tools that infants can use to interact with others vary from visual contact to smiling or vocalizing, and also include motor activity. However, surprisingly few studies have explored how the nature and context of social interactions affect infants’ engagement in motor activity.

**Methods:**

We investigated the kinematic properties of foot and face movements produced by 11 infants aged between 5 and 9 months during six contrasting dyadic episodes (i.e. passive presence of a stranger or the infant's mother, weak or intense interaction with the stranger/mother as she sings a nursery play song).

**Results:**

The infants’ face and foot motor activity was significantly reduced during the interactive episodes, compared with the episodes without any interaction, in both the mother and stranger conditions. Furthermore, the level of their motor activity was significantly lower in the stranger condition than in the mother one for some parameters.

**Conclusion:**

These results are in line with those reported by previous studies and confirm the relevance of using motor activity to delineate the early forms of interactive episodes in infants.

Over the past 50 years, a growing number of studies have focused on newborns’ social skills (e.g. Striano & Reid, 2006). In particular, these studies have explored infants’ abilities during dyadic interactions, focusing on (1) the influence of the partner and the interactive context on infants’ behavior and (2) the means by which infants take part in social interactions. Many studies have demonstrated that infants are sufficiently sensitive to identify their interlocutor, and to perceive regularities in the interaction (e.g. Bushnell, 2001). In early development, infants use a range of different *tools* to initiate, maintain, or put an end to social interactions, including visual contact, facial expressions, vocalizations, and body movements (e.g. Stern, 1974; Trevarthen, 1998; Tronick, 1989). The first three communication channels have been thoroughly explored in research on the early development of interactions.

Visual contact (i.e. eye-to-eye contact) during dyadic interactions is the first way that infants capture and maintain their partner's attention. Infants’ gaze behavior has been extensively studied and is known to play an important role in social development (e.g. Harel, Gordon, Geva, & Feldman, 2011; Messer & Vietze, 1984). Facial expressions (brow raising, brow knitting, smiling, etc.) also constitute the foundations of early interactions. Smiling is a positive facial expression that appears very early on in development, and is regarded as one of the first social signals (e.g. Wolff, 1987). Finally, vocalizations such as crying, cooing, or babbling remain one of the most important channels of adult–infant communication and have been shown to contribute strongly to early interactions (e.g. Locke, 2006). Most of the time, these tools are used in a redundant or coordinated way (e.g. Lewis & Coates, 1980; Miller & Commons, 2007). For instance, newborns express their disapproval of communication disturbance in the still-face paradigm through gaze aversion, showing a distressed face and crying (e.g. Nagy, 2008), and 6-month-olds through a greater amount of vocalization (Franklin et al., 2014). By 2 months of age, infants observed in usual contexts respond to their mother with clear signs of active engagement, such as smiling, cooing expressions, brow knitting, and gazing at her face (Lavelli & Fogel, 2013).

Body movements (e.g. gross motor movements) during early dyadic interactions have been explored less often. However, since Wallon's (1949–2002) seminal research, the assumption has been that newborns rely on their tonus and movements to express their emotional states. Thelen extensively analyzed motor development (Thelen & Smith, 1994). In a review focusing on rhythmical behavior, she argued that such rhythmical behavior initially functions like a non-specific motor response to a variety of eliciting stimuli, but gradually turns into instrumental behavior (Thelen, 1981). During interaction with the caregiver or social interaction with another adult, rhythmical behavior is a general, non-specific response to a variety of situations. For instance at this age, ‘the infant uses rhythmical kicking both to greet the mother and to protest the removal of a toy’ (Thelen, 1981, p. 241). The regular repetition of a movement, which characterizes rhythmical behaviors, is thought to have a cumulative effect on the observer and increase its potency for communication. On this point, Thelen (1981) suggested that rhythmical movement may progressively and opportunistically come to be used to communicate the infant's affect. This instrumental behavior is therefore molded from *wired-in* components and contributes to the founding of early social interaction. Trevarthen (1986) also attempted to delineate a theoretical background to *intersubjective motor control*. Within the context of intersubjectivity theory, Trevarthen (2011) argued that a child's head and limb movements convey emotions and are modulated by the experiences of being held and touched by the mother/caregiver. As a result, newborns ‘demonstrate self-regulations of movement publicly, for other persons, anticipating or provoking their response’ (p. 121). This author has carried out a great deal of research on the development of protoconversation and the coordination and synchronization of dyadic interactions, demonstrating the high precision of the exchanges that take place very early on (see Trevarthen, 2011; Trevarthen & Delafield-Butt, 2014).

Recent studies systematically exploring variations in movements during dyadic interactions are few and far between. They have featured a variety of methodologies, including visual inspection and Gestalt perception (e.g. Einspieler, Prechtl, Ferrari, Cioni, & Bos, 1997), applying an evaluation grid to the appearance or disappearance of motor activity (e.g. Nakata & Trehub, 2004), synthetic measurement of body movements (e.g. Robertson & Johnson, 2009), or kinematic analysis using video or 3D motion capture (e.g. Disselhorst-Klug, Heinze, Breitbach-Faller, Schmitz-Rode, & Rau, 2012). Thelen (1981) precisely described the frequency and context of occurrence of rhythmical stereotypies in a longitudinal study of infants observed in their homes from age 4 weeks to 1 year. Among other things, she demonstrated that infants’ motor repertoire is dominated by rhythmical movements in the middle of the first year. Furthermore, a large number of stereotypies can be clearly associated with changes in stimuli in social contexts (e.g. appearance of the caregiver, interruption in feeding, presentation of a toy). Watanabe, Homae, and Taga (2011) compared the body movements of 2- to 3-month-old infants in two conditions involving an overhead mobile. In one condition, the infant's arm was connected to the toy, whereas in the other condition an experimenter activated the toy. Their results revealed reduced velocity of body movements by the 3-month-old infants assigned to the *observer* role of looking at the mobile activated by the experimenter, compared with both the baseline and the *player* condition. The distinct observer behavior was indicative of a motor inhibition mechanism that is triggered when attention is required (Watanabe et al., 2011). In the same vein, Bacher and Robertson (2001) observed an inverse correlation between spontaneous fluctuations in body movement and visual inspection in 3-month-old infants exposed to visual stimuli. In the context of social situations, Nakata and Trehub (2004) observed a reduction in body movements in 6-month-old infants in the case of maternal singing compared with maternal speech. The reduction in body movements (i.e. disappearance of movement other than that of wrists and fingers) was concomitant with an increase in visual fixation. To sum up, despite Thelen's (1981) and Trevarthen's (1986) assertions that body movements are key to the study of early communication and its development, few studies have so far explored how infants engage in motor activity according to the nature and context of social interactions.

The aim of this preliminary study was twofold. First, we sought to develop analytical tools for studying infants’ motor activity in an ecological procedure that would be compatible with early dyadic interactions. The aim was to compare different measurements to verify the relevance of using motor activity to delineate the early forms of interactive episodes in infants. Second, we wanted to investigate the properties of infants’ motor activity during interactive episodes so as to further document their sensitivity to the characteristics of the interaction. More specifically, the present study examined the properties of foot and face movements produced by infants aged between 5 and 9 months during six contrasting dyadic episodes, plus a control episode when the infant was alone. These episodes varied with regard to the level of interaction (no interaction, weak interaction, intense interaction) and the nature of the social relationship (mother vs. stranger).

## Method

### Participants

Eleven healthy, full-term infants took part in the study. Their ages ranged between 5 and 9 months (mean age=6.9 months, SD=1.1 months; 4 boys and 7 girls). One additional infant took part in the study, but was excluded from the analyses because he produced very large movements throughout the task, playing with his legs and trunk. All the infants were Caucasian and came from middle-to-upper-class families living in a southern French city. Written consent was obtained from the parents of each infant. The study was carried out in accordance with the latest Declaration of Helsinki.

### Procedure

The individual sessions took place in a room either in a maternity hospital or in the university. Infants sat on a special chair which allowed them free movement of their legs, arms, and head, even though they were strapped in for safety. The procedure comprised six contrasting dyadic episodes, plus a control episode where the infant was alone, that each lasted 45 s (Table 1). All the children were exposed to the episodes in the same order because we wanted the child to be used to the setting before the mother left the room.

**Table 1:** Detailed description of the seven episodes to which the infants were exposed, according to partner and context

No.	Partner	Context	Description
1	Mother	Silent distant	The mother sits at a table reading a magazine about 2 m from her infant
2	Mother	Singing close	The mother comes close to the child and sings a play song with hand movements. This song (*Ainsi font font font les petites marionettes*) is one of the best known in France
3	Mother	Singing distant	The mother sits at the same table (about 2 m away) and sings the same play song, without any hand movements
4	Infant alone		The infant is left alone in the room. The mother is hidden behind a screen
5	Stranger	Silent distant	The stranger sits at the table (about 2 m away) reading a magazine
6	Stranger	Singing close	The stranger comes close to the child and sings the play song with the hand movements
7	Stranger	Singing distant	The stranger sits at the table (about 2 m away) and sings the play song without any hand movements

Each episode lasted 45 s.

### Material

**Fig. 1:**
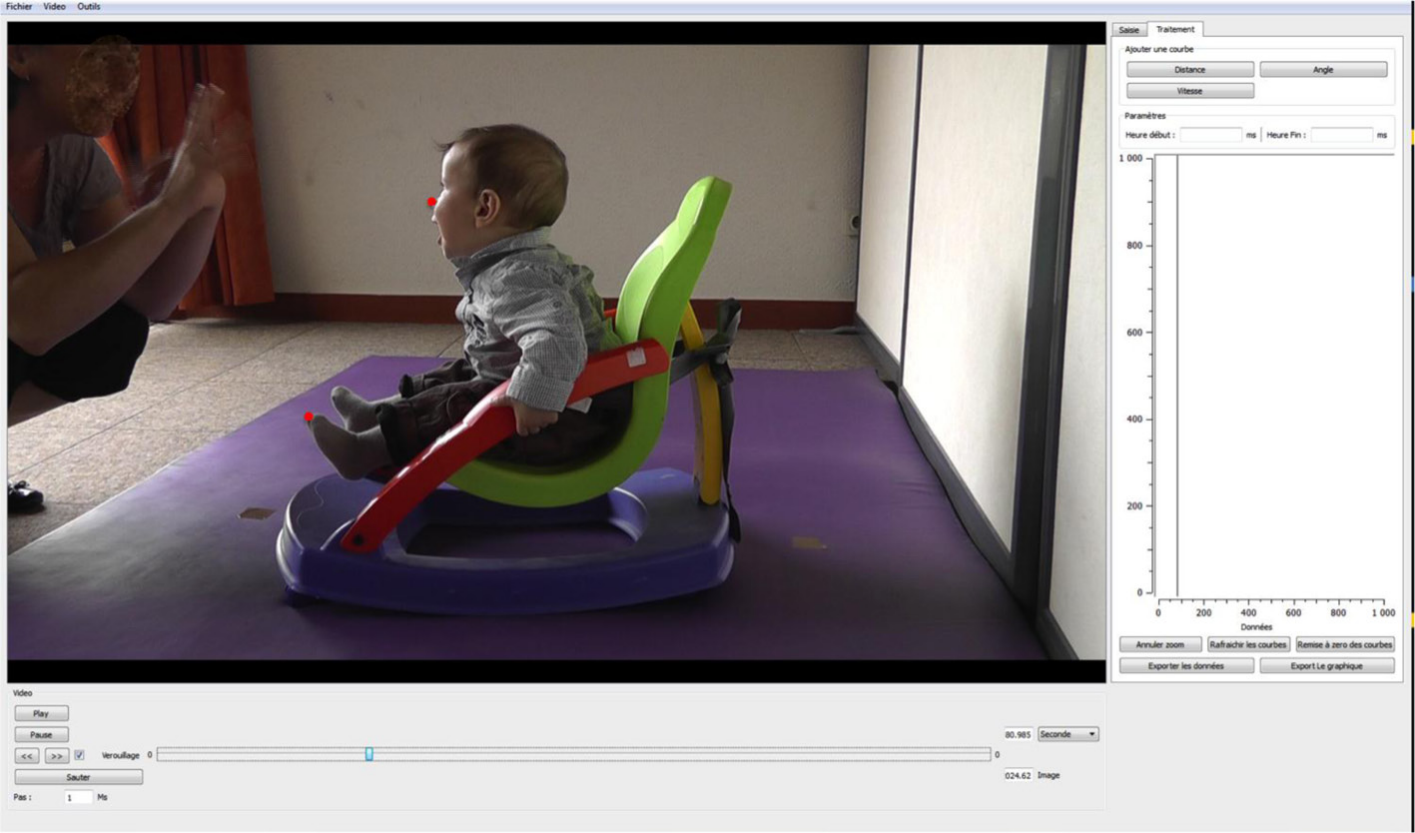
Video Analyser interface. In the present study, circle-shaped markers were positioned frame by frame on the child's nose and tip of the left toe. Graphs illustrated changes in the position of these landmarks during an 8-s sequence (5 Hz) in the middle of the interaction episode.

A camera to the left of the infant, positioned 2 m away, recorded the entire experiment. Anatomical landmark displacements during the interaction were measured with Video Analyser, a program developed in our laboratory (Mare, 2010). This program allows markers, manually positioned frame by frame, to be converted into x and y coordinates, thus providing distances of interest, as well as graphic illustrations of predefined variables (see Fig. 1 for an overview of the interface and see the Measures section for more details).

### Measures

Two anatomical landmarks were selected according to their relevance in describing movements of the head (nose) and movements of a distal part of the body (tip of left toe). The videos we recorded were analyzed offline, frame by frame. We positioned two circle-shaped markers on each frame within a sequence lasting 8 s at a frequency of 5 Hz. This sequence was selected in the middle of each interaction episode in order to limit the possible interferences of the previous condition or of the change itself. This choice appeared relevant to the assessment of differences between conditions because it provided the infants with sufficient time to ‘get into’ the new condition. We had the possibility of zooming in on the frame and adjusting the size of the markers for greater accuracy (Fig. 1). When one of the landmarks could not be found in a particular frame, the software allowed us to move on to the next frame to continue coding. Out of a total of 3,465 frames, 196 (5.7%) could not be coded for the nose landmark, and 27 (0.8%) for the toe landmark. In total, seven sequences, one per condition, were coded for each infant.

The x and y coordinates were then used to calculate the markers’ displacement (cm) and the tangential velocity (cm/s) of this displacement in the sagittal plane for each time interval (0.2 s). The infants’ *overall motor activity* (%) was calculated as the percentage of time intervals where the velocity of the markers was above 1 cm/s. Furthermore, we calculated the *mean velocity* (cm/s) and *utilization distribution* (cm^2^) of each marker. The mean velocity was calculated as the mean of the tangential velocities calculated for each time interval. The utilization distribution was calculated with the kernel method (Silverman, 1986). This method, borrowed from ethological studies, provides a bivariate probability density function that gives the probability of finding the marker at a particular location. We used the kernel utilization distribution function of the R-package adehabitatHR with a probability of 95%. Finally, we calculated the *coefficient of correlation* between the nose and toe displacements (cm) in each sequence. To avoid any artifact effect, we excluded every sequence where more than 15% of the data were missing (three sequences).

### Statistical analysis

Statistical analyses were conducted with the Statistica statistical package. Episode 4, where the infant was left alone in silence, was used as a baseline and compared with the other episodes with paired *t*-tests. Repeated-measures analyses of variance (ANOVA) were run on Episodes 1, 2, 3, 5, 6, and 7, with partner (mother vs. stranger) and context (silent distant, singing close, singing distant) as independent variables. We applied Tukey's HSD test as a *post hoc* analysis. Effect sizes were calculated, with partial eta-squared (abbreviated as $\eta_{p}^{2}$) representing the proportion of variability in the outcome measure that was attributable to a factor, all other sources of variance being partialed out (Cohen, 1973). The threshold for statistical significance was set at *p<*0.05.

## Results

### Overall motor activity

**Fig. 2:**
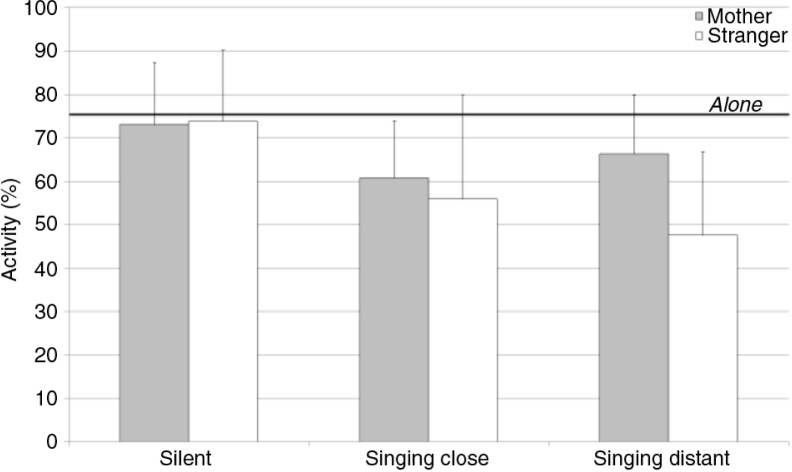
Mean values and standard deviations for the infants’ overall activity according to context and partner. The thick black line indicates the baseline value when the infants were left alone in the room.

The infants’ overall activity was about 75.45% (± 14.72) when alone (Fig. 2). This activity diminished significantly when the stranger sang at a distance, *t*(10)=5.18, *p<*0.001, or close by, *t*(10)=4.09, *p<*0.01, or when the mother sang close by, *t*(10)=2.64, *p<*0.05.

The ANOVA revealed a significant effect of context, *F*(2, 20)=6.04, *p<*0.01, $\eta_{p}^{2}=0.38$ . The *post hoc* Tukey test showed that both singing conditions induced a decrease in activity. Interestingly, this decrease was affected by partner, *F*(2, 20)=5.79, *p<*0.05, $\eta_{p}^{2}=0.37$: overall activity tended to be lower when the stranger sang at a distance than when the mother sang at a distance.

### Face displacements according to partner and context

**Fig. 3:**
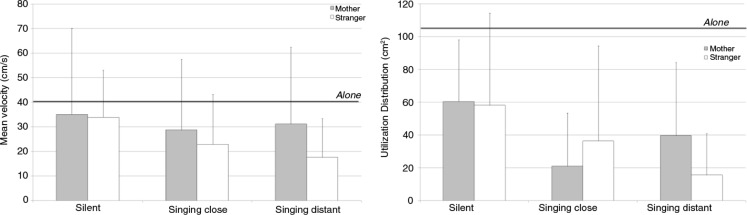
Mean values and standard deviations for the mean velocity (left) and utilization distribution (right) of the nose marker in the sagittal plane according to context and partner. The thick black line indicates the baseline value when the infants were left alone in the room.

The movement of the nose marker in the sagittal plane was about 40.152 cm/s (± 30.68) when the infants were left alone (Fig. 3). This activity was significantly lower when the stranger sang from a distance, *t*(10)=4.43, *p<*0.005 or close by, *t*(10)=4.21, *p<*0.005, or when the mother sang close by, *t*(10)=3.3, *p<*0.01.

The main effect of context was not significant, *F*(2, 20)=2.73, *p<*0.08, $\eta_{p}^{2}=0.21$ , but the velocity of the nose displacement was greater with the mother than with the stranger, *F*(1, 10)=5.57, *p<*0.05, $\eta_{p}^{2}=0.36$.

When we considered the utilization distribution of the nose marker (Fig. 3), the difference between the episode where the infants were left alone and the dyadic episodes never reached significance, probably because of the high level of interindividual variability when the infants were alone (mean 106.9,±167.5). However, context had a significant effect when the stranger or mother was in the room, *F*(2, 16)=8.96, *p<*0.005, $\eta_{p}^{2}=0.53$. The partner's singing induced a substantial reduction in the area of the nose marker's displacement. The Tukey *post hoc* test indicated that the differences were significant between both the silent and singing close by episodes (*p<*0.007) and the silent and singing at a distance episodes (*p<*0.005).

### Foot displacements according to partner and context

**Fig. 4:**
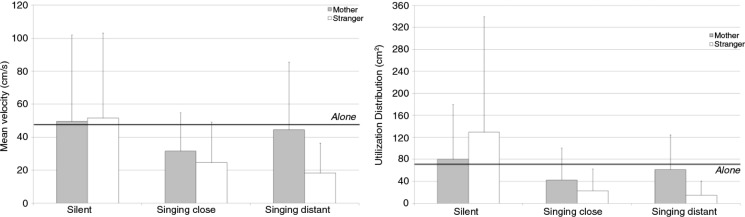
Mean values and standard deviations for the mean velocity (left) and utilization distribution (right) of the toe marker in the sagittal plane according to context and partner. The thick black line indicates the baseline value when the infants were left alone in the room.

The displacement of the toe marker in the sagittal plane was about 47.15 cm/s (± 22.85) when the infants were alone (Fig. 4). This activity was significantly lower when the stranger sang from a distance, *t*(10)=4.05, *p<*0.05, or close by, *t*(10)=3.07, *p<*0.05.

The main effect of context was significant, *F*(2, 20)=3.49, *p<*0.05, $\eta_{p}^{2}=0.26$, but the *post hoc* analysis only indicated a trend toward significance for the difference between the silent and singing close by episodes (*p*=0.058).

The utilization distribution of the toe marker was about 74.3±70.7 when the infants were alone (Fig. 4). It differed in the episodes when the mother, *t*(9)=2.73, *p<*0.05, or the stranger, *t*(9)=2.45, *p<*0.05, sang at a distance, or when the mother sang close to the infant, *t*(9)=3.01, *p<*0.05.

There was no significant main effect. A context×partner interaction indicated that the difference between the partners depended on the context, *F*(2, 16)=3.67, *p<*0.05, $\eta_{p}^{2}=0.29$. To sum up, the infants’ leg movements increased when the mother sang at a distance after singing close to the infant, and decreased when the stranger sang at a distance after singing close to the infant. The Tukey *post hoc* test indicated that the difference was only significant between the silent and stranger singing close by (*p<*0.05) and silent and stranger singing at a distance (*p<*0.01) episodes.

### Correlation between foot and face displacements

The correlation between the displacement of the toe and displacement of the nose varied between −0.33 and 0.69, depending on the infant and the episode (Table 2). The difference between Episode 4 (when the infant was alone) and each of the dyadic episodes was never significant, probably because of the high interindividual variability once again.

**Table 2:** Mean correlation coefficient (standard deviation) according to context and partner

	Mother	Stranger
Alone	0.07 (0.22)	
Silent	0.08 (0.15)	0.20 (0.20)
Singing distant	0.13 (0.27)	0.01 (0.12)
Singing close	0.26 (0.30)	0.20 (0.16)

The ANOVA revealed a significant effect of context, *F*(2, 16)=4.38, *p<*0.05, $\eta_{p}^{2}=0.35$. The *post hoc* Tukey test indicated that the two singing conditions differed significantly from each another (*p<*0.05), with a significantly higher correlation between foot and face displacement for singing close by. Table 3 summarizes the partial eta-squared statistics of all the comparisons

**Table 3:** Partial eta-squared statistics for each variable and each comparison

Variable	Partner	Context	Partner×context
Overall activity	0.18	0.38**	0.37*
Face–velocity	0.36*	0.21	0.14
Face–utilization distribution	0.07	0.53***	0.12
Foot–velocity	0.11	0.26*	0.10
Foot–utilization distribution	0.01	0.17	0.29*
Face–foot correlation	0.03	0.35*	0.18

* p<0.05.

** p<0.01.

*** p<0.005.

## Discussion

We used six parameters to characterize infants’ activity in contrasting episodes: overall activity, face–mean velocity, face–utilization distribution, foot–mean velocity, foot–utilization distribution, and face–foot correlation. We found that the overall motor activity of the infants’ faces and feet was significantly lower in the interactive episodes than in the episode where there was no interaction, in both partner conditions. Although there was a very high level of interindividual variability for all the motor activity parameters, some of the latter nonetheless differed significantly when the infants were in the presence of their mother rather than a stranger, demonstrating the relevance of our methodology. We set out the results below, according to the different points we addressed in this study.

### Sensitivity of the infants’ motor activity to the presence of a partner in the room and to the play song

The infants’ motor behavior when they were left alone in the room was highly variable. Some of them visually explored the environment, moving their head, legs, and body intensively, while others stayed still. Interindividual variability was therefore very high for all the parameters in this episode. In the two silent episodes, the main parameters were also variable, and the differences between the silent and alone episodes never reached significance. Nonetheless, we believe that rather than behaving in exactly the same way, the infants actually reduced their head movements slightly when the partner became visible. A larger group with more homogenous ages could help us to identify small variations such as these, which only appeared at the descriptive level in the present study.

Results revealed an overall reduction in motor activity when the mother or a stranger sang the play song to the child. As a whole, the infants tended to move less when the partner interacted with them rather than remaining silent. This reduction was visible at all levels of analysis: overall activity, face and foot, with facial immobility (reduced utilization distribution) being the most salient result. *Post hoc* analysis revealed a difference between the silent condition and the singing distant/close conditions for two parameters: overall motor activity and utilization distribution for head movements were significantly lower when the partner sang. This result confirms the considerable attractiveness of singing for infants and the positive impact of maternal singing on sustained interest and arousal (Nakata & Trehub, 2004; Trehub & Nakata, 2002), and extends this observation to the singing of a stranger. It is also in line with the notion that reduced body movements reflect selective attention paid to others (Robertson & Johnson, 2009).

Interestingly, the *post hoc* analysis applied to the correlation between foot and face revealed a difference between the distant and close singing conditions. Movements of the head and leg were more closely correlated in the close singing episodes than in the episodes where the partner sang at a distance. This correlation indicated that there was a concomitant reduction in both face and foot movements in the close singing condition. We had expected this correlation to be close in the silent condition, too, as the infants tried to establish an interaction or signal their satisfaction to a distant silent partner using their repertoire of motor stereotypies. There are two possible reasons why this was not the case. First, there was considerable interindividual variability during these two episodes: some infants explored their feet, some sucked their fingers, and others looked around the room. Second, Thelen (1981) observed that infants produce rhythmical movements whenever there is a change of stimulus, so this variable should be explored in a way that takes the time sequencing of tasks into account (e.g. Ramdani, Bouchara, & Caron, 2012).

### Sensitivity of the infants’ motor activity to the partner

The results of this preliminary study indicate that the presence of a partner (stranger or mother) modulated the motor activity produced by the infants. Overall motor activity tended to diminish when the stranger sang at a distance, whereas it tended to augment when the mother sang at a distance, rather than close by. This effect was also significant when we considered mean velocity for face movements and utilization distribution for foot movements. Considered in the light of the attention hypothesis (Robertson & Johnson, 2009), this result suggests that the infants were more interested in staring at the stranger than at their mother. Gredebäck, Fikke, and Melinder (2010) observed a stranger preference in gaze following, which emerged between 4 and 6 months: infants followed the gaze of a stranger more than the gaze of their mother. According to these authors, strangers who, by essence, are novel, may merit a higher level of attention than mothers. The infants in the present study may have demonstrated more sustained attention during the interactions with the stranger than during the interactions with their mothers. Another, non-exclusive, hypothesis is that the infants used their body movements in an instrumental way with their mothers, based on the assumption that strongly moving their legs and head would prompt her to sing for longer or bring her closer (Thelen, 1981). Finally, it is worth reminding that the mothers left the room during the interactive episodes with the stranger, and that an order effect might induce such differences. Although none of the infants was weeping when the mother left the room, knowing whether infants’ behavior with the stranger might be caused by separation anxiety required further investigation.

### Methodological considerations

The present preliminary study was intended to characterize infants’ motor activities during dyadic interactions. We selected the face as an indicator of visual attention, because the head constitutes a reference frame to stabilize for visual perception (Assaiante & Amblard, 1995), which is fundamental during face-to-face interactions. The foot was selected as a distal part of the body whose activity is known to be frequent and structured in newborns (Thelen & Fisher, 1983). Results indicated that these two parts of the body moved differently according to context and partner. However, a closer look at the size effects (Table 3) suggests that the head movements were more sensitive to the interactions than the leg movements. Foot movements were not, however, independent of face movements (see above). We can conclude that an indicator comparable to the one that Robertson and colleagues (Robertson et al., 2007; Robertson & Johnson, 2009) used, when they measured body movements from piezoelectric sensors placed in the back and bottom of an infant seat, is not enough to delineate the early forms of interaction and infants’ initiatives. The problem is that a composite indicator makes it impossible to differentiate between the origins of the infants’ movements (leg, head, or arm).

We set out to propose a different way of analyzing infants’ motor activity that does not call for any physical markers. We chose to use four parameters extracted from the video in the sagittal plane: mean velocity, utilization distribution, correlation between head and foot displacement, and overall motor activity. Results showed that these parameters were not equally sensitive to the experimental conditions. Overall motor activity appeared to be a robust indicator of body movement variations linked to context and partner. Mean velocity and utilization distribution were expected to describe two different dimensions of movement: Were they fast or slow, wide or restricted? These dimensions did not manifest the same sensitivity to the experimental conditions at the face and foot levels. For instance, the mean velocity of face displacement varied according to partner, and the mean velocity of foot displacement varied according to context. Although differences have been found in their functional significance in the assessment of neurological development (see Disselhorst-Klug, Heinze, Breitbach-Faller, Schmitz-Rode, & Rau, 2012), to our knowledge, movement parameters have not been demonstrated to depend differently on contextual variables such as interaction partner or level of interaction. Further research could help to document these aspects of motor activity, looking at which aspects of their motor activity infants modulate or invest in in the context of dyadic interactions.

An important aspect of our results is the very high variability of infants’ movements. Interindividual variability is a common feature of early development and more especially in motor development (e.g. Cioni, Ferrari, & Prechtl, 1989). This preliminary study did not mean to investigate the underlying developmental processes or the individual differences, but rather to show that infants’ motor activities changed with the context of social interaction. Identifying the individual factors that could explain the variability constitutes a further step in this field of knowledge. We assume that both internal (prematurity, child development, physiological state) and experiential (mother wellbeing, culture) factors shape the way by which infants rely on their motor activity to interact with their mother or with a stranger. Additionally, it seems relevant to look at the dyadic specificities in the building of interactive routines (e.g. Bigelow, 1998) because self- or interactive contingency are among the mechanisms of attachment formation (Beebe et al., 2010). Using a longitudinal design to consider these questions will be appropriate to give an insight into the possible causes of these interindividual differences.

To sum up, this preliminary study attests to the relevance of analyzing motor activity in order to understand the mechanisms at play during early interactions and delineate the contribution of movement to communication during interactive episodes involving infants. Results showed that video recordings constitute a useful way of exploring motor activity. Their non-intrusive nature means that they do not interfere with the ongoing interaction. This preliminary study had several limitations, and the results should, of course, be considered with caution. First of all, there were only 11 participants, and the ecological nature of the procedure reinforced interindividual variability. The spontaneous production of motor activity has been shown on many occasions to vary considerably between infants (e.g. Robertson & Johnson, 2009; Thelen, 1981). Furthermore, our experimental plan should have been better balanced to allow for a comparison between the location of the partner and the silent/singing condition. Finally, the use of more sophisticated modeling tools would have helped to link the displacement of the landmarks in space and time. Once these limitations have been resolved, we plan to explore the development of motor activity in dyadic interactions between the first weeks of life and the point at which infants acquire rudimentary language. At the same time, the development of motor activity during dyadic interactions in infants that were born preterm should help us gain a better understanding of some of the atypical features of their early communication (Harel et al., 2011).
